# Defects in Vascular Mechanics Due to Aging in Rats: Studies on Arterial Wave Properties from a Single Aortic Pressure Pulse

**DOI:** 10.3389/fphys.2017.00503

**Published:** 2017-07-13

**Authors:** Chun-Yi Chang, Ru-Wen Chang, Shu-Hsien Hsu, Ming-Shiou Wu, Ya-Jung Cheng, Hsien-Li Kao, Liang-Chuan Lai, Chih-Hsien Wang, Kuo-Chu Chang

**Affiliations:** ^1^Department of Emergency Medicine, Taipei Veterans General Hospital Hsin-Chu, China; ^2^Department of Physiology, College of Medicine, National Taiwan University Taipei, China; ^3^Department of Emergency Medicine, National Taiwan University Hospital Taipei, China; ^4^Department of Internal Medicine, National Taiwan University Hospital Taipei, China; ^5^Department of Anesthesiology, National Taiwan University Hospital Taipei, China; ^6^Department of Surgery, National Taiwan University Hospital Hsin-Chu, China; ^7^Department of Surgery, National Taiwan University Hospital Taipei, China

**Keywords:** aging, aortic input impedance, arterial wave property, vascular impulse response, wave reflection factor, wave transit time

## Abstract

Changes in vascular mechanics due to aging include elevated vascular impedance, diminished aorta distensibility, and an accelerated return of pulse wave reflection, which may increase the systolic workload on the heart. Classically, the accurate measurement of vascular mechanics requires the simultaneous recording of aortic pressure and flow signals. In practice, it is feasible to estimate arterial wave properties in terms of wave transit time (τ_w_) and wave reflection index (RI) by using aortic pressure signal alone. In this study, we determined the τ_*w*_ and magnitudes of the forward (∣*P*_*f*_∣) and backward (∣*P*_*b*_∣) pressure waves in Long–Evans male rats aged 4 (*n* = 14), 6 (*n* = 17), 12 (*n* = 17), and 18 (*n* = 24) months, based on the measured aortic pressure and an assumed triangular flow (*Q*^tri^). The pulsatile pressure wave was the only signal recorded in the ascending aorta by using a high-fidelity pressure sensor. The base of the unknown *Q*^tri^ was constructed using a duration, which equals to the ejection time. The timing at the peak of the triangle was derived using the fourth-order derivative of the aortic pressure waveform. In the 18-month-old rats, the ratio of τ_*w*_ to left ventricular ejection time (LVET) decreased, indicating a decline in the distensibility of the aorta. The increased ∣*P*_*b*_∣ associated with unaltered ∣*P*_*f*_∣ enhanced the RI in the older rats. The augmentation index (AI) also increased significantly with age. A significant negative correlation between the AI and τ_*w*_/LVET was observed: AI = −0.7424 − 0.9026 × (τ_*w*_/LVET) (*r* = 0.4901; *P* < 0.0001). By contrast, RI was positively linearly correlated with the AI as follows: AI = −0.4844 + 2.3634 × RI (*r* = 0.8423; *P* < 0.0001). Both the decreased τ_*w*_/LVET and increased RI suggested that the aging process may increase the AI, thereby increasing the systolic hydraulic load on the heart. The novelty of the study is that *Q*^tri^ is constructed using the measured aortic pressure wave to approximate its corresponding flow signal, and that calibration of *Q*^tri^ is not essential in the analysis.

## Introduction

Aging is known to be associated with deterioration in many structural and functional properties of aortas and large arteries, including dilated vessel diameter, increased wall thickness, diminished wall elasticity, and endothelial dysfunction (Lakatta and Yin, 1982; O'Rourke and Nichols, 2005). As age advances, the following histological alterations in the vasculature are observed: an increased rate of endothelial cell apoptosis, the degeneration of smooth muscles in the media, fragmentation of and decrease in the content of elastic fibers, and an increase in the number of irregularly arranged collagen fibers in the stroma (Lakatta, 1979; Yin, 1980; Lakatta and Yin, 1982; Chang et al., 1998). Collagen crosslinking by non-enzymatic glycation is also enhanced within the arterial wall (Sims et al., 1996; Schleicher et al., 1997). All these factors contribute to the age-related changes in the mechanical properties of the vasculature, including elevated arterial impedance, diminished aorta distensibility, and an accelerated return of pulse wave reflection (O'Rourke and Nichols, 2005). These changes in vascular mechanics are accelerated in the incidence of hypertension (Najjar et al., 2005; Franklin, 2006), coronary heart diseases (Mattace-Raso et al., 2006), congestive heart failure (Sutton-Tyrrell et al., 2005), and stroke (O'Leary et al., 1999) withadvancing age.

The physical properties of the arterial system are reflected in the aortic input impedance (*Z*_*i*_), which is the aortic pressure-flow relation in the frequency domain (McDonald, 1974; O'Rourke, 1982; Milnor, 1989; Wang et al., 2014a). While the aortic characteristic impedance (*Z*_*c*_) is known (Nichols and O'Rourke, 2011), the wave separation method can be derived in the time domain to resolve the measured aortic pressure wave into its forward (*P*_*f*_) and backward (*P*_*b*_) components (Westerhof et al., 1972). The arterial wave transit time (τ_*w*_) can be computed using the impulse response function, which is the time-domain equivalent of its input impedance in the frequency domain (Laxminarayan et al., 1978; Sipkema et al., 1980). Thus, the accurate measurement of arterial wave properties, including arterial τ_*w*_ and wave reflection magnitude (RM) or wave reflection index (RI), requires the simultaneous recording of aortic pressure and flow signals.

In practice, it is feasible to estimate the τ_*w*_ and magnitudes of the forward and backward pressure waves by using aortic pressure signal alone. Westerhof et al. (2006) provided a novel method to calculate the pressure wave reflection by using only the measured aortic pressure. Replacing the unknown flow signal with a triangular wave shape (*Q*^tri^), they successfully resolved the measured aortic pressure wave into its components, *P*_*f*_ and *P*_*b*_, to calculate the RM or RI. Chang et al. (2017) elaborated this concept by determining the arterial τ_*w*_ through vascular impulse response analysis. They discovered that the aortic impulse response is an effective method for the estimation of arterial τ_*w*_ by using a single pressure pulse recording with an assumed *Q*^tri^.

In this study, we determined the age-related changes in arterial wave properties on the basis of the aortic pressure alone in Long–Evans male rats. The pulsatile pressure wave was the only signal recorded in the ascending aorta by using a high-fidelity pressure sensor. The timing at the peak of the *Q*^tri^ was derived using the fourth-order derivative of the aortic pressure waveform (Westerhof et al., 2006; Chang et al., 2017). On the basis of the measured aortic pressure and an assumed *Q*^tri^, we calculated the arterial τ_*w*_, magnitudes of the *P*_*f*_ and *P*_*b*_ waves, and augmentation index (AI) to delineate the age-related changes in the pulsatile component of the left ventricular (LV) afterload. The novelty of the study is that *Q*^tri^ is constructed using the measured aortic pressure wave to approximate its corresponding flow signal, and that calibration of *Q*^tri^ is not essential in the analysis.

## Methods

### Animals and catheterization

The effects of the aging process on the arterial mechanics were evaluated in specific pathogen-free Long–Evans male rats, aged 4 (*n* = 14), 6 (*n* = 17), 12 (*n* = 17), and 18 (*n* = 24) months. The rats were obtained from the colony maintained in the barrier facilities at the Laboratory Animal Center of the College of Medicine, National Taiwan University (Chang et al., 1998). All rats were allowed free access to Purina chow and water and were housed under 12 h light–dark cycles. The cages of the rats were examined periodically. Furthermore, the body weight (BW) of the rats was measured regularly to ensure the appropriate administration of the food. The experiment was conducted according to the *Guide for the Care and Use of Laboratory Animals*, and our study protocol was approved by the Animal Care and Use Committee of National Taiwan University (Chang et al., 1998).

The general surgical procedures and measurement of the cardiovascular variables in anesthetized rats were conducted as described previously (Chang et al., 1998). In brief, each rat was anesthetized with sodium pentobarbital (50 mg kg^−1^, I.P.), placed on a heating pad, intubated, and ventilated using a rodent respirator (model 131; New England Medical Instruments, Medway, MA, USA) (Wu et al., 2011). A high-fidelity pressure catheter (model SPC 320, size 2 French; Millar Instruments, Houston, TX, USA) was used to measure the pulsatile ascending aortic pressure via the isolated carotid artery of the right side. The lead II ECG was recorded using a Gould ECG/Biotach amplifier (Cleveland, OH, USA). Selective aortic pressure signals from 5 to 10 beats were averaged in the time domain, using the peak R wave of the ECG as a fiducial point.

**Figure 1:**
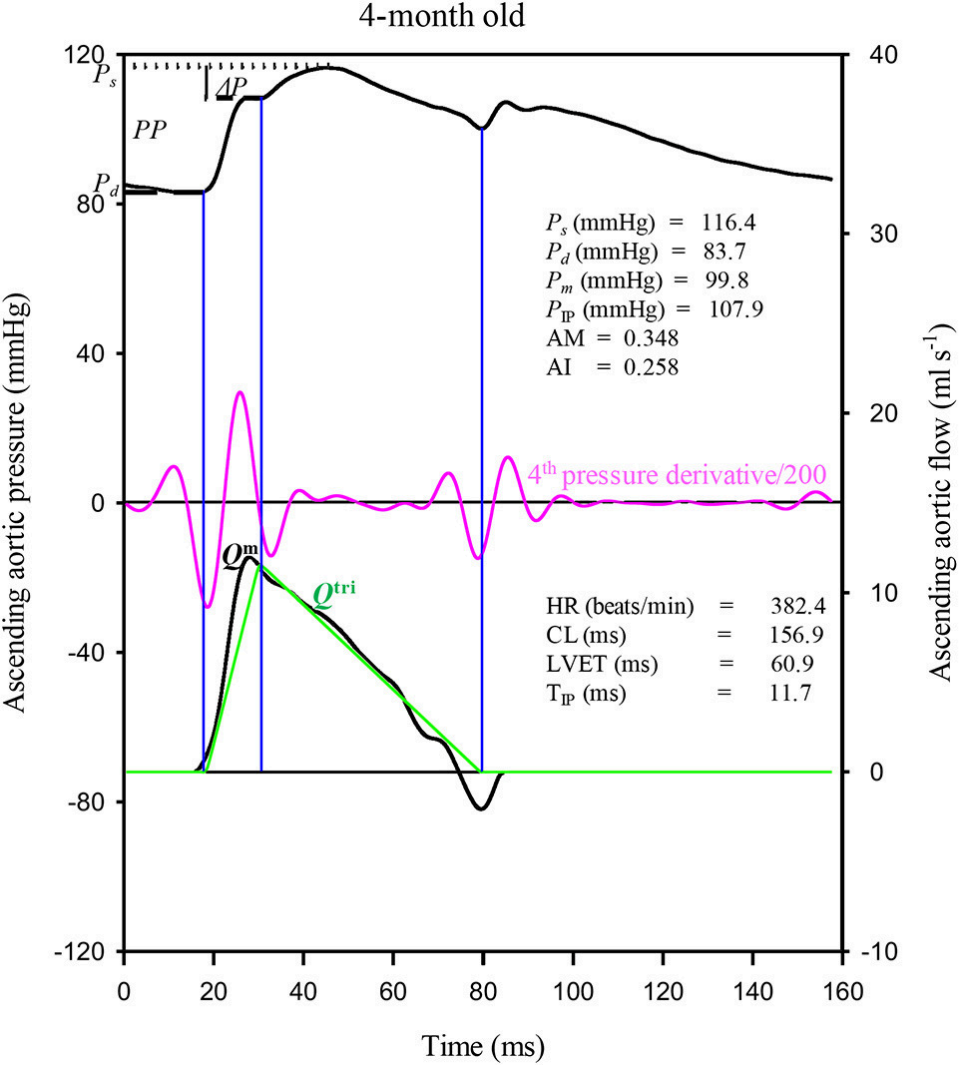
Construction of an uncalibrated triangular flow (*Q*^tri^) from the measured aortic pressure waveform in one rat aged 4 months as an example. The pressure and flow (*Q*^m^) signals were simultaneously recorded in the ascending aorta under the anesthetized, open-chest condition. AM, augmentation magnitude, given by Δ*P*/(*PP* − Δ*P*); AI, augmentation index, given by Δ*P*/*PP*; CL, cardiac cycle length; HR, basal heart rate; LVET, left ventricular ejection time; *P*_*s*_, systolic pressure; *P*_d_, diastolic pressure; *P*_*m*_, mean pressure; *PP*, pulse pressure; *P*_IP_, pressure at inflection point; Δ*P*, given by *P*_*s*_ − *P*_IP_; T_IP_, time to inflection point.

To show the similarity between the measured and predicted arterial wave properties, the aortic pressure and flow (*Q*^m^) signals were also simultaneously recorded in one rat aged 4 months, as an example, under the anesthetized, open-chest condition (Figure 1). The chest was opened through the second intercostal space on the right side. The pulsatile *Q*^m^ waveform was measured by using an electromagnetic flow probe (100 series, internal circumference 8 mm, Carolina Medical Electronics, King, NC, USA), which was positioned around the ascending aorta (Chang et al., 2017).

### Construction of the unknown flow wave by using a triangle

The unknown *Q*^tri^ was derived from the pressure waveform measured in the ascending aorta (Westerhof et al., 2006; Chang et al., 2017). The onset and termination of LV ejection were identified as the intersection of two tangential lines near the foot of the pressure wave (the first vertical blue lines in Figures 1, **3A,B**) and near the incisura (the third vertical blue lines in Figures 1, **3A,B**), respectively (Chang et al., 2015). The base of the *Q*^tri^ was constructed using a duration set equal to the ejection time, which is the time difference between the start and end points. The timing at the peak of the triangle was derived from the fourth-order derivative of the aortic pressure wave (the pink curves in Figures 1, **3A,B**; Westerhof et al., 2006; Chang et al., 2017). After ejection commenced, the first zero-crossing curve from above to below (the second vertical blue lines in Figures 1, **3A,B**) determined the peak of the triangle of blood flow, which was the inflection point of the pressure wave (Kelly et al., 1989; Westerhof et al., 2006; Chang et al., 2017). Thus, the uncalibrated *Q*^tri^ was approximated by a triangular shape (the green curves in Figures 1, **3A,B**) and represented the corresponding flow wave of the aortic pressure signal.

After identifying the inflection point, the augmentation of pressure (Δ*P*) can be defined as the difference between the systolic pressure (*P*_*s*_) and the pressure at the inflection point (*P*_IP_): Δ*P* = *P*_*s*_ − *P*_IP_ (Figures 1, **3A,B**; Westerhof et al., 2006). The difference between *P*_*s*_ and the diastolic pressure (*P*_*d*_) is the pulse pressure (*PP* = *P*_*s*_ − *P*_*d*_). Thus, the augmentation magnitude (AM) is defined as the ratio of Δ*P* to the initial pressure rise (*PP* − Δ*P*), given by AM = Δ*P*/(*PP* − Δ*P*) (Westerhof et al., 2006). The AI is the pressure augmentation (Δ*P*) divided by the total pressure amplitude (*PP*), given by AI = Δ*P*/*PP*.

### Impulse response function curve

**Figure 2:**
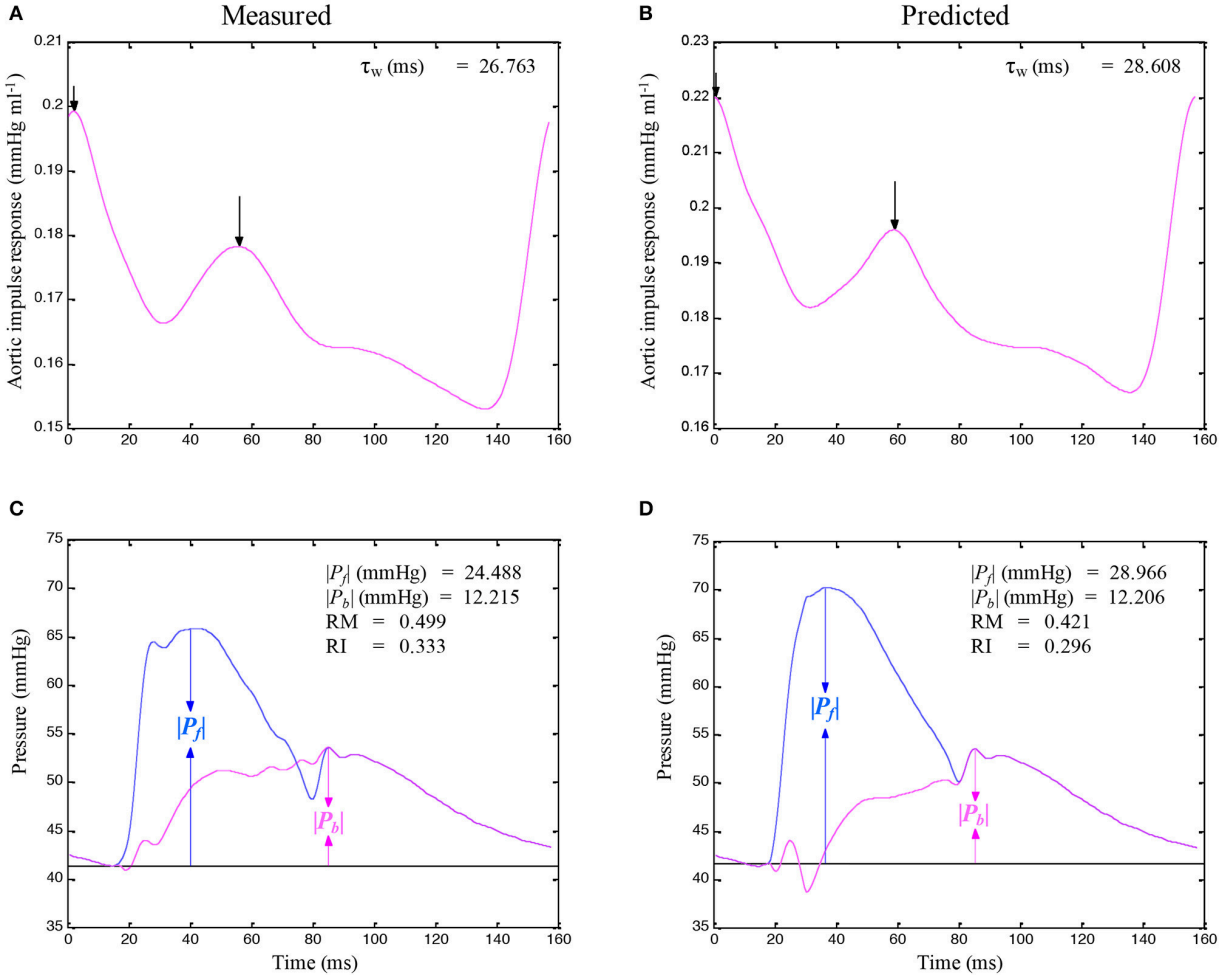
Similarity between the measured aortic impulse response obtained from the measured pressure and *Q*^m^ **(A)** and the predicted aortic impulse response obtained from the measured pressure and *Q*^tri^ **(B)** in the same rat, which is shown in Figure 1. The measured *P*_*f*_ and *P*_*b*_ waves **(C)** and the predicted *P*_*f*_ and *P*_*b*_ waves **(D)** are also depicted. In (**C,D**), the amplitudes (peak - trough) of the *P*_*f*_ and *P*_*b*_ are represented by ∣*P*_*b*_∣ and ∣*P*_*f*_∣, respectively. *P*_*f*_, forward pressure wave; *P*_*b*_, backward pressure wave; RM, wave reflection magnitude, given by ∣*P*_*b*_∣/∣*P*_*f*_∣; RI, wave reflection index, given by ∣*P*_*b*_∣/(∣*P*_*f*_∣ + ∣*P*_*b*_∣); *Q*^m^, measured aortic flow; *Q*^tri^, uncalibrated triangular flow; τ_*w*_, wave transit time.

A standard Fourier series expansion technique was performed to calculate the *Z*_*i*_ from the ratio of the ascending aortic pressure harmonics to the corresponding flow harmonics from either *Q*^m^ or *Q*^tri^ (McDonald, 1974; Milnor, 1989; Nichols and O'Rourke, 2011; Chang et al., 2015). The *Z*_*c*_ was calculated by averaging the high-frequency moduli of the *Z*_*i*_ data points from 4 to 10 harmonics (Wang et al., 2014a). The arterial τ_*w*_ was computed using the impulse response function curve (the pink lines in Figures 2A,B, **4A,B**; Sipkema et al., 1980; Latson et al., 1987), which was generated by using an inverse Fourier transformation of the *Z*_*i*_ after multiplying the first 12 harmonics by a Dolph–Chebychev weighting function with order 24 (Laxminarayan et al., 1978; Chang et al., 2015). One-half of the time difference between the appearance of the second reflected peak (long arrow) and the initial peak (short arrow) in the impulse response curve approximates the τ_*w*_ in the lower body circulation (Laxminarayan et al., 1978; Sipkema et al., 1980; Wu et al., 2012).

### Arterial wave separation analysis

The following equations were used to calculate the *P*_*f*_ and *P*_*b*_ from the measured aortic pressure wave (*P*_*ao*_) in the time domain (Murgo et al., 1981; Chang et al., 2015):

(1) $$P_{f}(t)=\frac{P_{ao}(t)+Z_{c}\times Q(t)}{2}$$

(2) $$P_{b}(t)=\frac{P_{ao}(t)-Z_{c}\times Q(t)}{2}$$

The calculations of the *P*_*f*_ and *P*_*b*_ by using *Q*(*t*) from either *Q*^m^ or *Q*^tri^ are depicted in Figures 2C,D, **4C,D**. The amplitudes (peak - trough) of the *P*_*b*_ and *P*_*f*_ are represented by ∣*P*_*b*_∣ and ∣*P*_*f*_∣, respectively. The aortic RM was then defined as the ratio of ∣*P*_*b*_∣ and ∣*P*_*f*_∣ (i.e., RM = ∣*P*_*b*_∣/∣*P*_*f*_∣; Westerhof et al., 2006). The reflection index (RI) was calculated as RI = ∣*P*_*b*_∣/(∣*P*_*f*_∣ + ∣*P*_*b*_∣).

### Statistics

Results are expressed as means ± standard error. A one-way analysis of variance (ANOVA) was performed to determine the statistical significance of the results for multiple comparisons of the effect of the aging process on arterial wave properties (Wu et al., 2012). Statistical significance was assumed at the level of *P* < 0.05. In cases where the ANOVA results indicated that a hemodynamic variable differed significantly among age groups, Tukey's honest significant difference method was used to determine the groups of rats that exhibited divergent mean values for that variable (Wang et al., 2014b).

## Results

Table 1 presents the effect of age on BW, basal heart rate (HR), cardiac cycle length (CL), LV ejection time (LVET), and aortic pressure profile. In the rats, a significant increase in BW, decrease in HR, increase in CL, and prolongation in LVET were observed with an increase in age. However, the BW, HR, CL, and LVET did not significantly differ between the rats aged 12 and 18 months. The systolic blood pressure (*P*_*s*_) did not change significantly as animals aged; however, diastolic (*P*_*d*_), mean (*P*_*m*_), mean systolic (*P*_*ms*_), and mean diastolic (*P*_*md*_) aortic pressures, and the *P*_*ms*_/*P*_*md*_ ratio were significantly lower in 18-month-old rats than in the rats from other age groups.

**Table 1:** Basic hemodynamic data measured in rats aged 4, 6, 12, and 18 months.

	**BW**	**HR**	**CL**	**LVET**	***P_*s*_***	***P_*d*_***	***P_*m*_***	***P_*ms*_***	***P_*md*_***	***P_*ms*_*/*P_*md*_***
**AGE (MONTHS)**										
4 (*n* = 14)	374.1 ± 6.1	383.2 ± 6.1	157.1 ± 2.4	63.4 ± 0.7	139.7 ± 3.9	115.4 ± 2.7	128.8 ± 3.2	134.0 ± 3.5	125.2 ± 3.0	1.070 ± 0.005
6 (*n* = 17)	431.2 ± 8.9	372.8 ± 6.4	161.8 ± 2.9	66.7 ± 1.3	139.6 ± 3.5	116.9 ± 3.3	129.8 ± 3.3	134.7 ± 3.3	126.4 ± 3.3	1.066 ± 0.004
12 (*n* = 17)	482.1 ± 10.5	324.6 ± 9.2	187.5 ± 6.0	77.3 ± 1.8	139.1 ± 3.4	115.2 ± 2.6	128.2 ± 2.9	133.0 ± 3.1	124.8 ± 2.8	1.066 ± 0.003
18 (*n* = 24)	490.2 ± 9.3	307.9 ± 7.7	197.6 ± 4.8	82.4 ± 1.9	130.2 ± 3.1	100.9 ± 2.3	116.0 ± 2.6	122.2 ± 2.7	111.5 ± 2.4	1.096 ± 0.004
***P*****-VALUE**										
4 vs. 6	<0.001	NS	NS	NS	NS	NS	NS	NS	NS	NS
4 vs. 12	<0.001	<0.001	<0.001	<0.001	NS	NS	NS	NS	NS	NS
4 vs. 18	<0.001	<0.001	<0.001	<0.001	NS	<0.005	<0.05	<0.05	<0.01	<0.001
6 vs. 12	<0.005	<0.001	<0.001	<0.001	NS	NS	NS	NS	NS	NS
6 vs. 18	<0.001	<0.001	<0.001	<0.001	NS	<0.001	<0.01	<0.05	<0.01	<0.001
12 vs. 18	*NS*	NS	NS	NS	NS	<0.005	<0.05	<0.05	<0.01	<0.001

*All values are expressed as means ± standard error. BW, body weight (g); HR, heart rate (beats min^−1^); CL, cardiac cycle length (ms); LVET, left ventricular ejection time (ms); P_s_, systolic pressure (mmHg); P_d_, diastolic pressure (mmHg); P_m_, mean aortic pressure (mmHg); P_ms_; mean systolic pressure (mmHg); P_md_, mean diastolic pressure (mmHg); NS, not significant (P > 0.05)*.

Table 2 presents the pressure characteristics in relation to the pulse wave reflection derived from the aortic pressure signal in rats of different ages. Rats aged 18 months had markedly higher T_IP_ than did the rats aged 4 and 6 months. However, *P*_IP_ significantly decreased and Δ*P* increased in the 18-month-old rats compared with those in rats from other age groups. The *PP* values were markedly higher in the 18-month-old rats than were those values in the 4-, 6-, and 12-month-old rats. Moreover, rats aged 18 months had significantly higher ∣*P*_*b*_∣, but not ∣*P*_*f*_∣ than did the rats in other age groups. Although, the arterial τ_*w*_ did not change significantly as animals aged, the τ_*w*_/CL ratio was significantly lower in the rats aged 18 months than in the rats aged 4 and 6 months but not in those aged 12 months. No correlation between arterial τ_*w*_ and T_IP_ was observed.

**Table 2:** Characteristics in relation to pulse wave reflection derived from aortic pressure waveform in rats aged 4, 6, 12, and 18 months.

	**T_IP_**	***P*_IP_**	***PP***	**Δ*P***	**∣*P_f_*∣**	**∣*P_b_*∣**	***τ_*w*_***	***τ_*w*_*/CL**
**AGE (MONTHS)**								
4 (*n* = 14)	11.9 ± 0.4	129.2 ± 3.0	24.3 ± 1.5	10.4 ± 1.1	17.7 ± 1.0	10.9 ± 0.6	21.9 ± 1.3	0.140 ± 0.008
6 (*n* = 17)	11.5 ± 0.3	129.9 ± 3.1	22.8 ± 1.0	9.7 ± 0.8	16.4 ± 0.6	10.5 ± 0.4	21.7 ± 0.6	0.134 ± 0.003
12 (*n* = 17)	13.3 ± 0.5	127.5 ± 2.9	23.9 ± 1.1	11.6 ± 0.8	16.3 ± 0.8	10.9 ± 0.5	23.9 ± 0.6	0.129 ± 0.004
18 (*n* = 24)	14.6 ± 0.5	115.2 ± 2.6	29.4 ± 1.3	15.0 ± 0.9	18.2 ± 0.7	13.1 ± 0.6	22.5 ± 0.4	0.115 ± 0.003
***P*****-VALUE**								
4 vs. 6	NS	NS	NS	NS	NS	NS	NS	NS
4 vs. 12	NS	NS	NS	NS	NS	NS	NS	NS
4 vs. 18	<0.001	<0.01	<0.05	<0.005	NS	<0.05	NS	<0.005
6 vs. 12	NS	NS	NS	NS	NS	NS	NS	NS
6 vs. 18	<0.001	<0.01	<0.005	<0.001	NS	<0.005	NS	<0.05
12 vs. 18	NS	<0.05	<0.01	<0.05	NS	<0.005	NS	NS

*All values are expressed as means ± standard error. T_IP_, Time to inflection point (ms); P_IP_, pressure at the inflection point (mmHg); PP, pulse pressure (mmHg); ΔP, P_s_ − P_IP_ (mmHg); ∣P_f_∣, magnitude of forward pressure wave (mmHg); ∣P_b_∣, magnitude of reflected pressure wave (mmHg); τ_w_, arterial wave transit time (ms); NS, not significant (P > 0.05)*.

Figure 1 illustrates the construction of an uncalibrated *Q*^tri^ from the measured pressure waveform in one rat aged 4 months as an example. The pressure and *Q*^m^ signals were simultaneously recorded in the ascending aorta under the anesthetized, open-chest condition. Figure 2 depicts the similarity between the measured aortic impulse response obtained from the measured pressure and *Q*^m^ (A) and the predicted aortic impulse response obtained from the measured pressure and *Q*^tri^ (B) in the same rat. The measured *P*_*f*_ and *P*_*b*_ waves (C) and the predicted *P*_*f*_ and *P*_*b*_ waves (D) are also presented. Although, the *Q*^tri^ shape is an approximation that may differ from the actual *Q*^m^ shape, this approximation gave results close to those obtained with the measured *Q*^m^.

**Figure 3:**
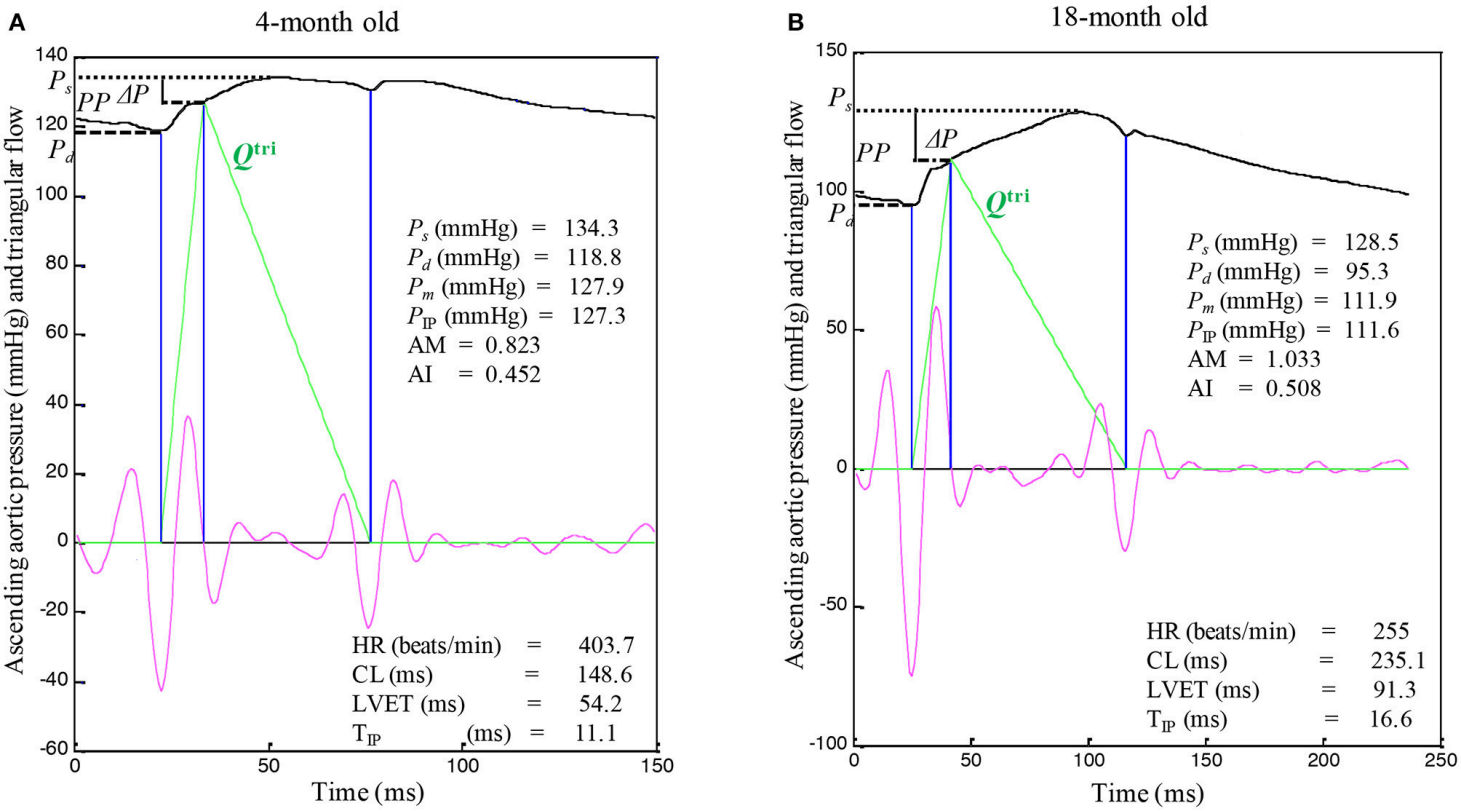
An uncalibrated triangular flow (*Q*^tri^) derived from the measured aortic pressure wave in rats aged 4 **(A)** and 18 months **(B)**. The 4-month old rat is the same one as shown in Figure 1. Only the pressure signal was recorded in the ascending aorta under the anesthetized, closed-chest condition. AM, augmentation magnitude, given by Δ*P*/(*PP* − Δ*P*); AI, augmentation index, given by Δ*P*/*PP*; CL, cardiac cycle length; HR, basal heart rate; LVET, left ventricular ejection time; *P*_*s*_, systolic pressure; *P*_*d*_, diastolic pressure; *P*_*m*_, mean aortic pressure; *PP*, pulse pressure; *P*_IP_, pressure at inflection point; Δ*P*, given by *P*_*s*_ − *P*_IP_; T_IP_, time to inflection point.

**Figure 4:**
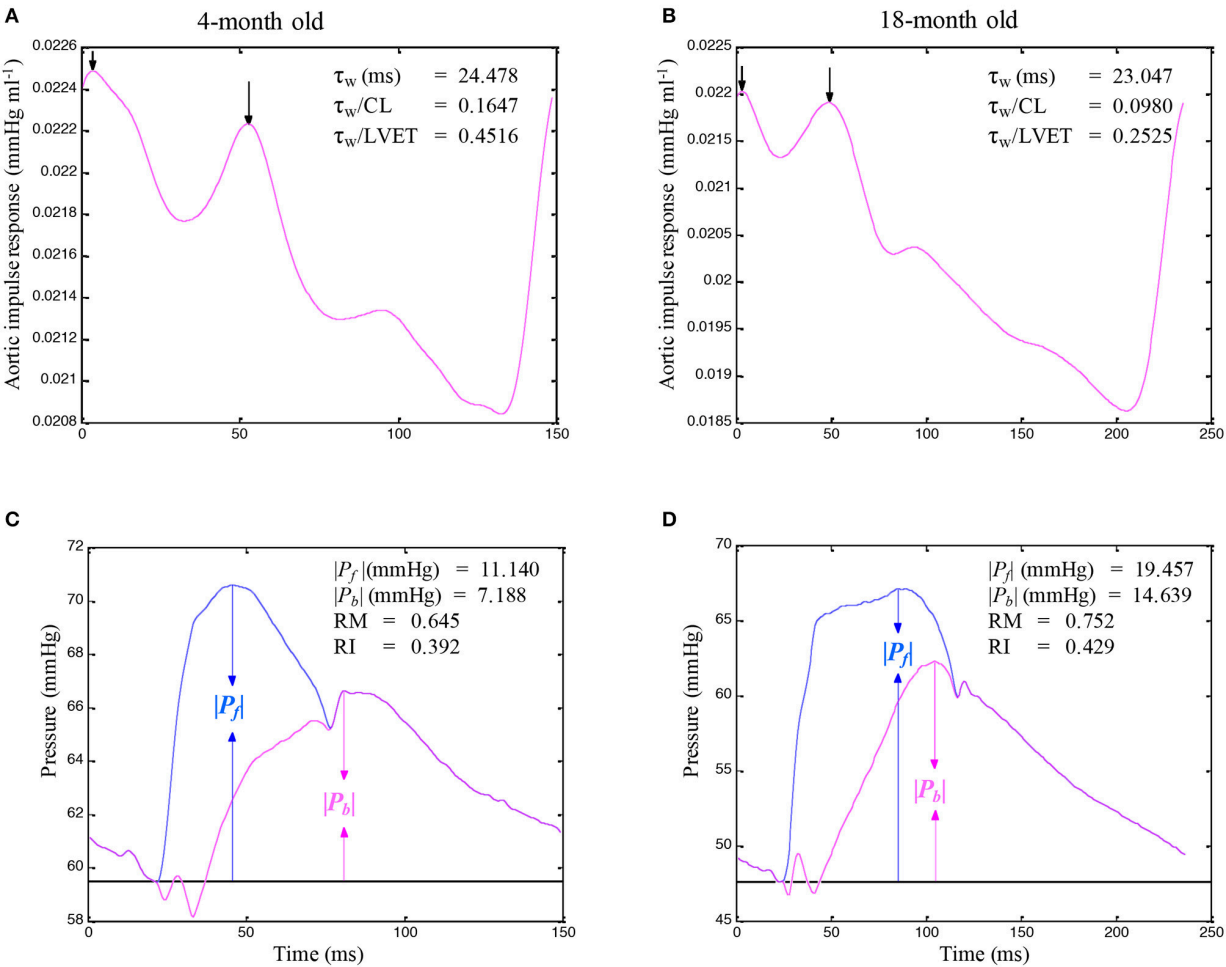
Impulse response function curve obtained from the measured aortic pressure and an assumed triangular flow (*Q*^tri^) in the same rats aged 4 **(A)** and 18 **(B)** months, which are shown in Figure 3. The *P*_*f*_ and *P*_*b*_ waves for the corresponding rats aged 4 **(C)** or 18 **(D)** months are also depicted. In **C** and **D**, the amplitudes (peak - trough) of the *P*_*f*_ and *P*_*b*_ are represented by ∣*P*_*b*_∣ and ∣*P*_*f*_∣, respectively. CL, cardiac cycle length; LVET, left ventricular ejection time; *P*_*f*_, forward pressure wave; *P*_*b*_, backward pressure wave; RM, wave reflection magnitude, given by ∣*P*_*b*_∣/∣*P*_*f*_∣; RI, wave reflection index, given by ∣*P*_*b*_∣/(∣*P*_*f*_∣ + ∣*P*_*b*_∣); τ_*w*_, wave transit time.

Figures 3A,B illustrate the construction of an uncalibrated triangular flow from the measured pressure waveform in 4- and an 18-month old rats, respectively. The rat aged 4 months was the same one as shown in Figure 1. The pressure wave was the only signal recorded in the ascending aorta under the anesthetized, closed-chest condition. Figure 4 depicts the aortic impulse responses obtained from the measured aortic pressure and *Q*^tri^ in the same 4- and 18-month old rats (Figures 4A,B, respectively). The *P*_*f*_ and *P*_*b*_ waves for the corresponding rats aged 4 (Figure 4C) or 18 (Figure 4D) months are also depicted.

**Figure 5:**
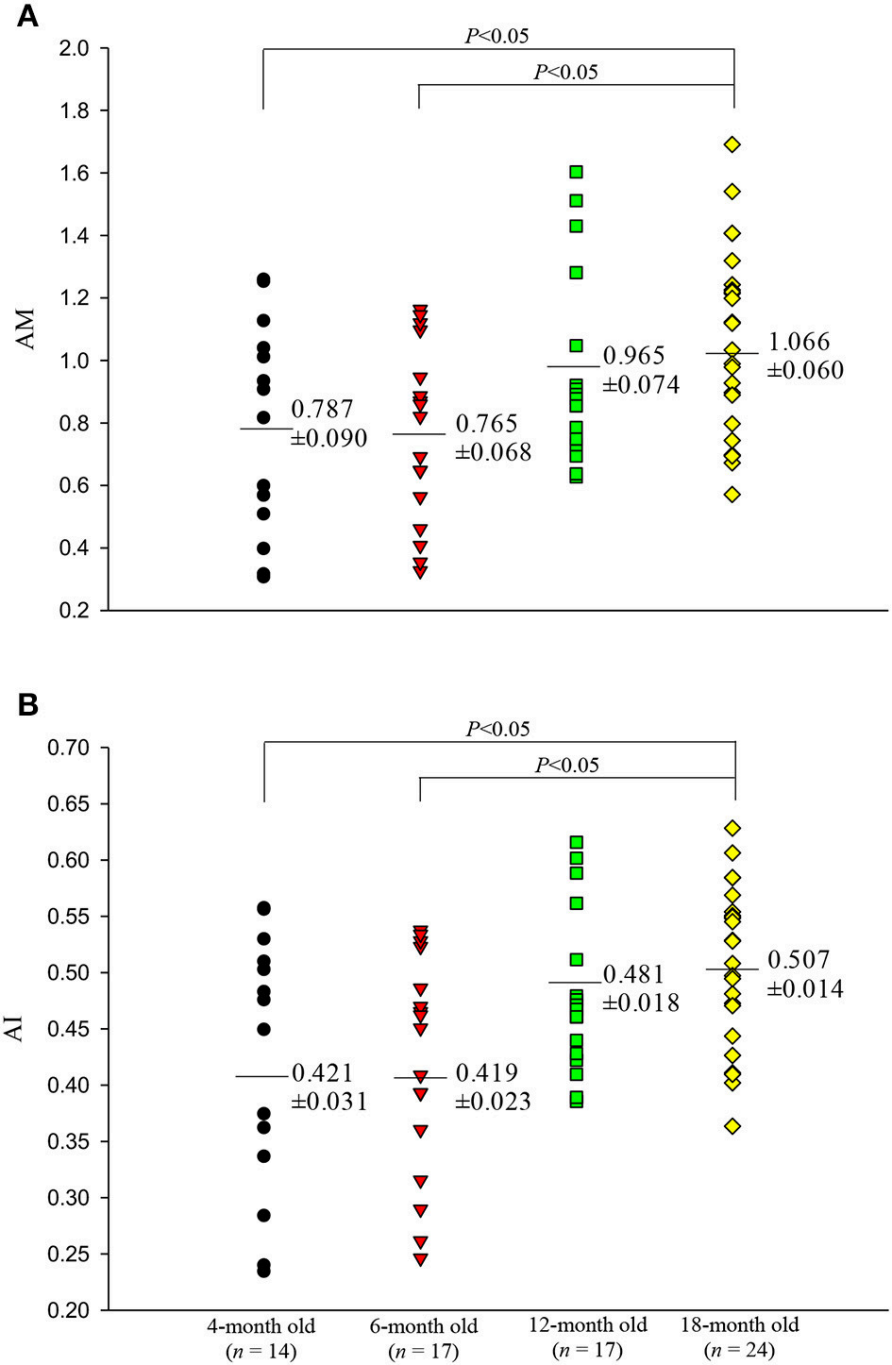
Effects of aging on augmentation magnitude AM **(A)** and augmentation index AI **(B)**. The rats aged 18 months had significantly higher AM and AI than did those aged 4 and 6 months.

**Figure 6:**
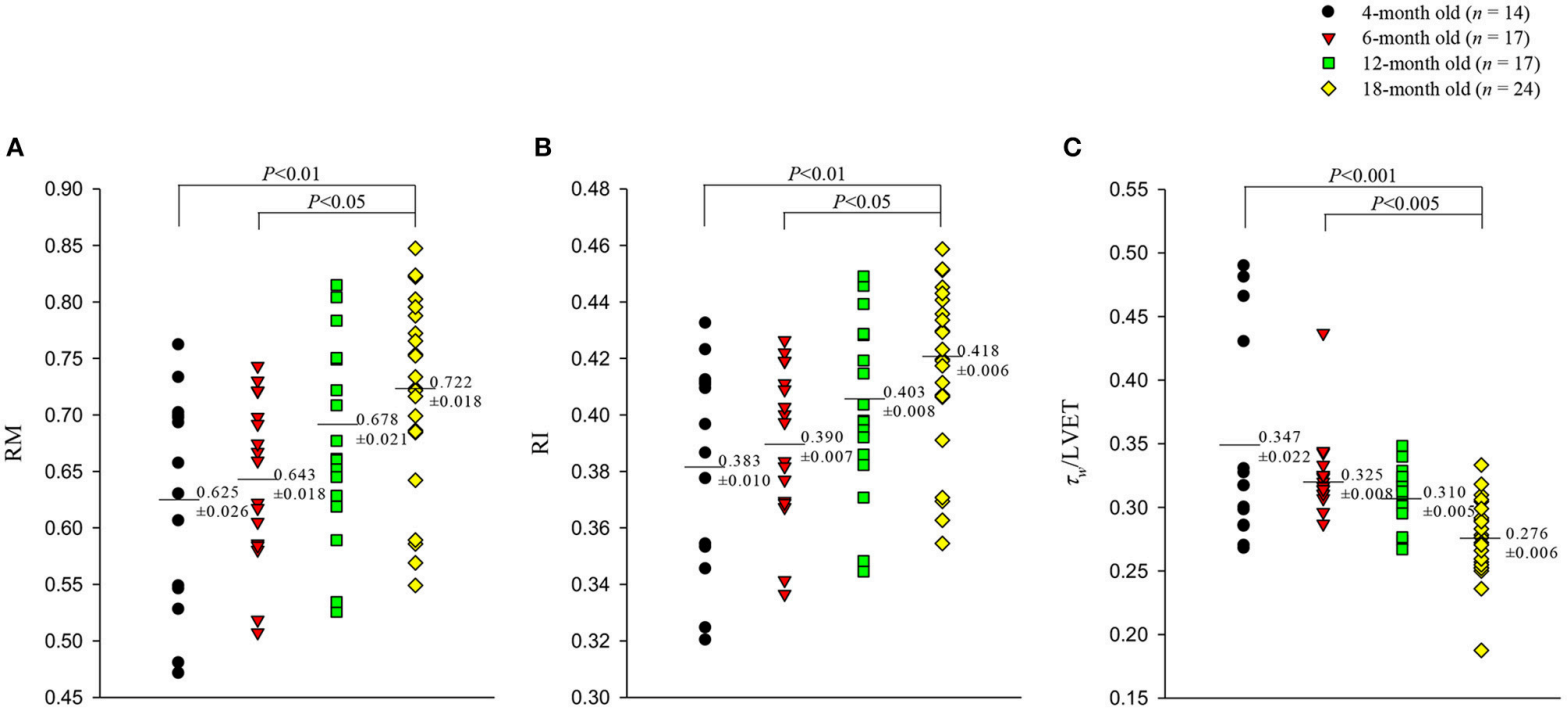
Effects of aging on wave reflection magnitude RM **(A)**, wave reflection index RI **(B)**, and the ratio of wave transit time (τ_*w*_) to left ventricular ejection period (LVET) **(C)**. The rats aged 18 months had significantly higher RM and RI than did those aged 4 and 6 months. Moreover, the τ_*w*_/LVET ratio was markedly lower in the 18-month-old rats than in the 4- and 6-month-old rats.

Figures 5, 6 illustrate the effect of aging on the arterial wave properties in terms of the AM, AI, RM, and RI as well as the τ_*w*_/LVET ratio. The AM (Figure 5A) and AI (Figure 5B) increased markedly in the 18-month-old rats compared with those in the 4- and 6-month-old rats. Moreover, the rats aged 18 months had significantly higher RM (Figure 6A) and RI (Figure 6B) values than did the rats aged 4 and 6 months. Although, the arterial τ_*w*_ did not change significantly as the rats aged (Table 2), the τ_*w*_/LVET ratio was markedly lower in the 18-month-old rats than in the 4- and 6-month-old rats (Figure 6C).

**Figure 7:**
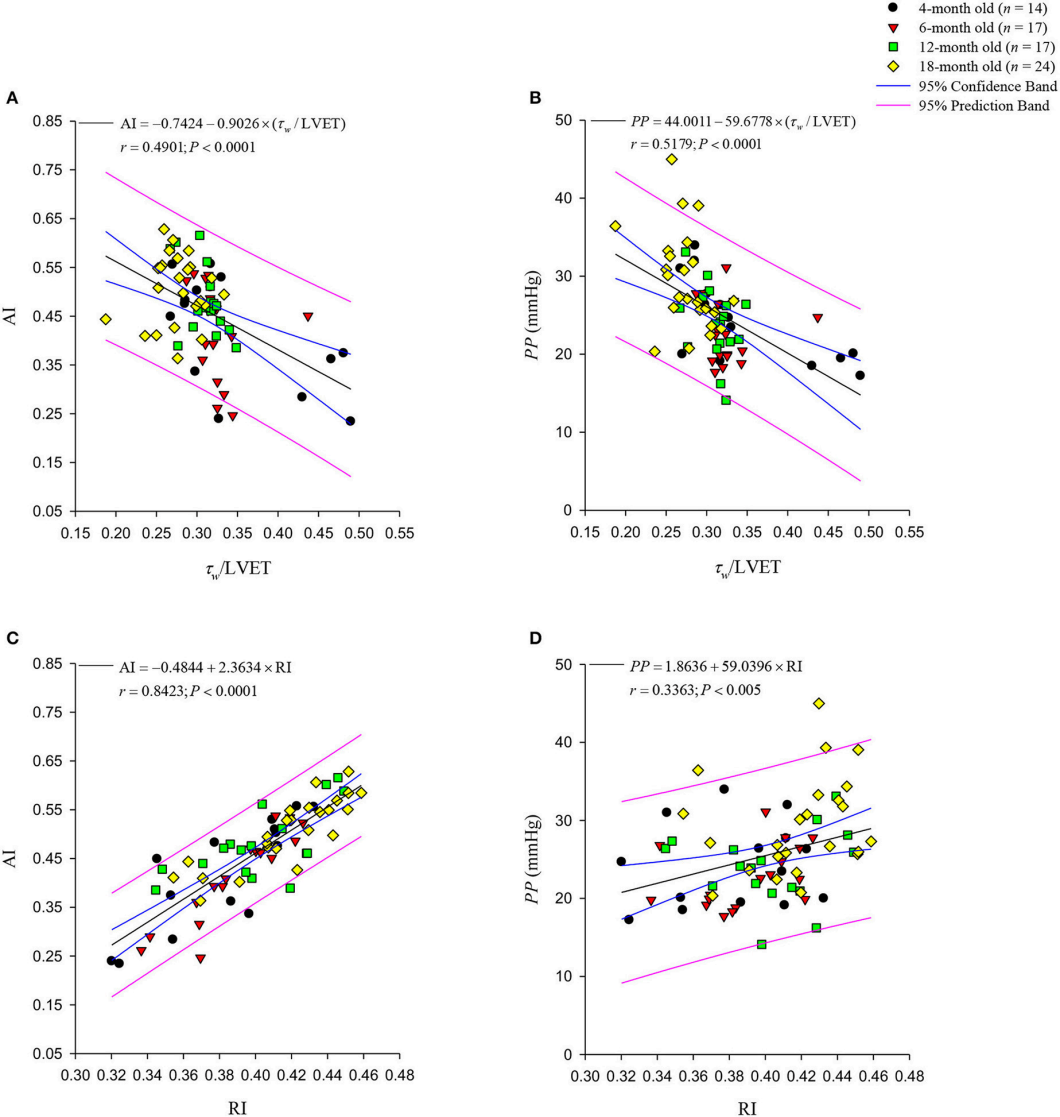
Relationships between the augmentation index (AI) and the ratio of wave transit time (τ_*w*_) to left ventricular ejection time (LVET) **(A)** and wave reflection index RI **(C)**. The aortic AI was significantly inversely related to the τ_*w*_/LVET ratio. By contrast, the RI and the aortic AI were directly related. In **(B)**, the pulse pressure (*PP*) shows a negative linear correlation with the τ_*w*_/LVET ratio. The positive regression line of *PP* with RI is depicted in **(D)**. The aortic AI had a closer correlation with the RI than the *PP* did.

Figure 7A illustrates relation between AI and τ_*w*_/LVET, and Figure 7C depicts that between AI and RI, which were calculated from the measured aortic pressure and an assumed *Q*^tri^. Figure 7A exhibits a significant inverse regression line for AI: AI = −0.7424 − 0.9026 × (τ_*w*_/LVET) (*r* = 0.4901; *P* < 0.0001). The regression equation of AI is given by AI = −0.4844 + 2.3634 × RI (*r* = 0.8423; *P* < 0.0001), and it is provided in Figure 7C. Figure 7B depicts relationship between *PP* and τ_*w*_/LVET, and Figure 7D presents that between *PP* and RI. Figure 7B depicts an inverse regression line given by *PP* = 44.0011 − 59.6778 × (τ_*w*_/LVET) (*r* = 0.5179; *P* < 0.0001). The regression equation of *PP* is *PP* = 1.8636 + 59.0396 × RI (*r* = 0.3363; *P* < 0.005), and it is provided in Figure 7D.

**Figure 8:**
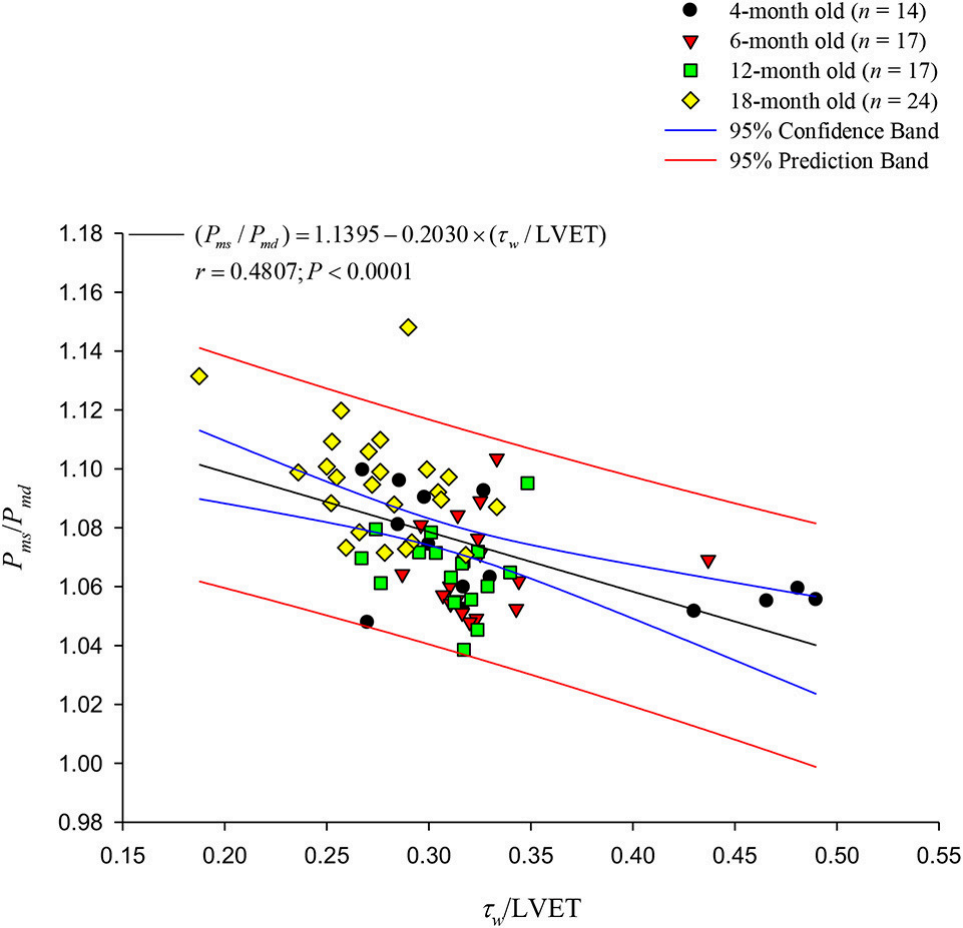
Relationship between the *P*_*ms*_/*P*_*md*_ ratio and the τ_*w*_/LVET ratio. The aortic *P*_*ms*_/*P*_*md*_ ratio was significantly inversely related to the τ_*w*_/LVET ratio. LVET, left ventricular ejection time; *P*_*md*_, mean diastolic pressure; *P*_*ms*_, mean systolic pressure; τ_*w*_, wave transit time.

Figure 8 illustrates relation between *P*_*ms*_/*P*_*md*_ and τ_*w*_/LVET, which exhibits a significant inverse regression line for *P*_*ms*_/*P*_*md*_: (*P*_*ms*_/*P*_*md*_) = 1.1395 − 0.2030 × (τ_*w*_/LVET) (*r* = 0.4807; *P* < 0.0001). However, the *P*_*ms*_/*P*_*md*_ ratio has no correlation with the RI.

## Discussion

Earlier studies on the age-related changes of arterial mechanical properties in different species have shown that considerable changes occur in response to age. In humans, Gundel et al. (1981) measured the aortic pressure and flow signals to calculate *Z*_*i*_ and *Z*_*c*_, demonstrating no relationship between age and *Z*_*c*_. By contrast, Nichols et al. (1985) reported that aging process increased the steady and pulsatile components of the hydraulic load and the arterial wave reflections returned earlier with increasing age. However, Cox (1977) measured the carotid elastic modulus in rats of different ages, and suggested that the aging heart was somewhat compensated by a decrease in hydraulic load. In the present study, the older rats exhibited increased arterial stiffness and magnitude of the reflected pressure wave, which enhanced the systolic workload on the heart and contributed to a mismatch between the myocardial oxygen demand andsupply.

In this study, the older rats exhibited a decline in HR and a prolongation in LVET. The decline in HR with age has also been described in other studies on rats (Bunag et al., 1990; Bunag and Teravainen, 1991). In the current study, significant declines in *P*_*d*_ and *P*_*m*_ but not *P*_*s*_ were observed in the 18-month-old rats. Although not reaching statistical significance, the rats aged 18 months had lower *P*_*s*_ than did the rats in the other age groups. The age-induced decline in the aortic pressure profile in the rats was consistent with the results of a previous report by Bunag et al. (1990).

The pulse wave velocity and traveling distance of pressure waves to reflection sites are the determinants of arterial τ_*w*_. In this study, we analyzed the aortic impulse response to calculate the arterial τ_*w*_ and found that the calculation of arterial τ_*w*_ was influenced by the CL (Figures 4A,B). Although the arterial τ_*w*_ did not change significantly as animals aged (Table 2), the τ_*w*_/CL ratio was significantly lower in the rats aged 18 months than in the rats aged 4 and 6 months but not in those aged 12 months. The decreased τ_*w*_/CL ratio indicated a decline in the distensibility of the aorta in rats with advancing age. In the absence of any significant change in the ∣*P*_*f*_∣, the significant rise in the ∣*P*_*b*_∣ in senescence (Table 2) was responsible for the increased RM (Figure 6A) and RI (Figure 6B). Both the arterial RM and RI augmented by age in turn increased the intensity of the wave reflection from the peripheral circulation in the older rats.

For the calculation of AM and AI, defining an inflection point on the aortic pressure waveform is imperative (Westerhof et al., 2006; London and Pannier, 2010). In this study, the inflection point was identified by the first zero-crossing curve from positive to negative on the fourth derivative of the pressure signal during ventricular ejection (Kelly et al., 1989; Westerhof et al., 2006; Chang et al., 2017). With the inflection point determined, we found that the rats aged 18 months had higher Δ*P* values than did those in other age groups. Although *PP* also increased as the rats aged, the increased Δ*P* dominated the increased *PP*, leading to an augmentation in aortic AM and AI.

From the definition, AI depends on the overlap between the *P*_*f*_ and the *P*_*b*_, which is determined by both the timing and magnitude of the reflected pressure wave (Westerhof et al., 2006; London and Pannier, 2010). The overlap between the *P*_*f*_ and the *P*_*b*_ depends on both the arterial τ_*w*_ and the LVET duration (London and Pannier, 2010). With a shortened τ_*w*_, the reflected waves return earlier, thereby affecting the central arteries during systole rather than diastole. With a lengthened LVET, it is favorable for the reflected wave to return during systole. Thus, the decreased τ_*w*_/LVET ratio may increase the overlap between the *P*_*f*_ and the *P*_*b*_, thereby increasing the systolic workload on the heart and reducing aortic pressure during diastole (London and Pannier, 2010).

In the present study, the τ_*w*_/LVET ratio was markedly smaller in the 18-month-old rats than in the rats from other age groups (Figure 6C). We found that the aortic AI was inversely affected by the arterial τ_*w*_/LVET ratio; thus, the lower the arterial τ_*w*_/LVET ratio, the higher the aortic AI (Figure 7A), which was consistent with those of a previous report by London and Pannier (2010). Although aging did not affect ∣*P*_*f*_∣, the older rats had increased ∣*P*_*b*_∣ (Table 2). Using simple linear regression analysis, we found that the aortic AI augmented by age was associated with the increased ∣*P*_*b*_∣: AI = 0.2423 + 0.0192 × ∣*P*_*b*_∣ (*r* = 0.5044; *P* < 0.0001), and had strong positive correlation with the RI (Figure 7C). As the arterial τ_*w*_/LVET ratio decreased and the arterial RI increased with age, the aortic AI increased, thereby augmenting the systolic workload on the heart.

Similarly to AI, *PP* was augmented as arterial τ_*w*_/LVET ratio decreased (Figure 7B) and arterial RI was increased (Figure 7D) in older rats. However, AI exhibited closer correlation with RI than *PP* did. These findings suggest that the aortic AI might be a better index describing the arterial wave properties than the aortic *PP*.

In considering the ventricular/vascular coupling, O'Rourke et al. (1984) suggested that the ascending aortic pressure wave includes two components: *P*_*ms*_, which is relevant to LV performance as a pump, and *P*_*md*_, which is relevant to LV perfusion. Thus, the augmented *P*_*ms*_/*P*_*md*_ ratio may cause a mismatch between the myocardial oxygen demand and supply. In this study, the rats aged 18 months had lower *P*_*ms*_ than did the rats in the other age groups (Table 1). Although *P*_*md*_ also decreased as the rats aged, the decreased *P*_*md*_ dominated the decreased *P*_*ms*_, leading to an increase in *P*_*ms*_/*P*_*md*_ ratio. The *P*_*ms*_/*P*_*md*_ ratio augmented by age was associated with the diminished τ_*w*_/LVET ratio (Figure 8), suggesting that as aging stiffened aortas, the older rats were accompanied with deterioration in the myocardial oxygen demand/supply ratio.

This study has several limitations. Because *Z*_*i*_ cannot be measured in conscious animals, evaluating the effects of pentobarbital-induced anesthesia on rats is impossible. The results reported here pertain only to the measurements made in anesthetized rats (Wu et al., 2012). This condition might have induced changes in the aortic pressure profiles and introduced reflex effects that are not observed under ordinary conditions (Wang et al., 2014a). The degree to which anesthesia influences the pulsatile hemodynamics in rats is not known with certainty. However, studies on other animals suggest that the effects are small in relation to the biological and experimental variability between animals (Cox, 1974). Moreover, the uncalibrated *Q*^tri^ was constructed using the measured aortic pressure wave to approximate the corresponding flow signal. Although the *Q*^tri^ is an approximation that may differ from the actual flow wave shape (Westerhof et al., 2006), the use of this concept to describe the arterial wave properties has been validated in studies by Westerhof et al. (2006) and Chang et al. (2017).

## Conclusions

We determined the mechanical defects due to arterial aging on the basis of the measured aortic pressure and an assumed triangular flow. Because the ∣*P*_*f*_∣ was unaltered, the increase in ∣*P*_*b*_∣ enhanced the intensity of the wave reflection, thereby augmenting RI in the older rats. A reduction in the τ_*w*_/CL ratio with age indicated a decline in the distensibility of the aorta, which resulted in arterial wave reflections returned earlier with increasing age. As the arterial τ_*w*_/LVET ratio decreased and arterial RI increased with age, the aortic AI increased. With an increase in the *P*_*ms*_/*P*_*md*_ ratio, the older rats were accompanied with deterioration in the myocardial oxygen demand/supply ratio. All these findings suggest that aging potentially impairs the pulsatile component of arterial mechanics, thereby increasing the systolic workload imposed on the heart.

## Perspectives

Our contribution in this endeavor is to provide a path to consider the clinical application of the method estimating the arterial wave properties, based on the measured pressure alone. The advantage of the technique is that an assumed *Q*^tri^ is derived from the measured pressure and that the flow calibration is not essential in the analysis. Westerhof et al. (2006) suggested that the method can also be performed using the carotid pressure wave as a surrogate for the pressure measured in the ascending aorta. The carotid pressure can be obtained non-invasively by applanation tonometry (Van Bortel et al., 2001) or by using a transfer function on finger arterial pressure (Westerhof, 2005) or radial artery pressure (Chen et al., 1997). In large epidemiological studies, it is helpful to evaluate the arterial wave properties as a function of age by using a minimally non-invasive measurement on aortic pressure alone, because the construction of the unknown *Q*^tri^, the separation of the aortic pressure waves, and the calculation of the arterial τ_*w*_ can be automated.

## Author contributions

CC, RC, CW, and KC developed the concept of the study, designed the experiments, and wrote the manuscript. CC, RC, and SH performed the animal experiments, collected the data, and performed statistical analysis. MW, YC, HK, and LL provided advice on the surgical procedures used in the study. CW and KC interpreted the data, supervised this work, and critically revised the manuscript. All authors have read and approved the final manuscript.

### Conflict of interest statement

The authors declare that the research was conducted in the absence of any commercial or financial relationships that could be construed as a potential conflict of interest.

## Abbreviations

AI: Augmentation index
AM: augmentation magnitude
CL: cardiac cycle length (ms)
HR: basal heart rate (beats min^−1^)
LVET: left ventricular ejection time (ms)
*Q*^m^: measured aortic flow wave (ml s^−1^)
*Q*^tri^: uncalibrated triangular flow wave (ml s^−1^)
*P*_*b*_: backward pressure wave (mmHg)
*P*_*d*_: diastolic aortic pressure (mmHg)
*P*_*f*_: forward pressure wave (mmHg)
*P*_IP_: pressure at inflection point (mmHg)
*P*_*m*_: mean aortic pressure (mmHg)
*P*_*md*_: mean diastolic pressure (mmHg)
*P*_*ms*_: mean systolic pressure (mmHg)
*PP*: pulse pressure (mmHg)
*P*_*s*_,: systolic aortic pressure (mmHg); Δ*P, P*_*s*_ − *P*_IP_ (mmHg)
RM: wave reflection magnitude
RI: wave reflection index
T_IP_: time to inflection point (ms)
*Z*_*c*_: aortic characteristic impedance (mmHg s ml^−1^)
*Z*_*i*_: aortic input impedance (mmHg s ml^−1^)
τ_*w*_: wave transit time (ms).

## References

[B1] Bunag R. D. Krizsan D. Eriksson L. (1990). Mediation of reflex tachycardia becomes exclusively 3-adrenergic in old Fischer 344 rats. Mech. Aging Dev. 52, 179–194. 2157925 10.1016/0047-6374(90)90123-w

[B2] Bunag R. D. Teravainen T. L. (1991). Waning cardiovascular responses to adrenergic drugs in conscious aging rats. Mech. Aging Dev. 61, 313–326. 10.1016/0047-6374(91)90063-6 1795569

[B3] Chang K. C. Tsai Y. F. Chow C. Y. Peng Y. I. Chen T. J. (1998). Age-related changes of arterial mechanical properties in rats: analysis using exponentially tapered T-tube model. J. Gerontol. Biol. Sci. 53A, B274–B280. 10.1093/gerona/53a.4.b274 18314557

[B4] Chang R. W. Chang C. Y. Lai L. C. Wu M. S. Young T. H. Chen Y. S.. (2017). Determining arterial wave transit time from a single aortic pressure pulse in rats: vascular impulse response analysis. Sci. Rep. 7:40998. 10.1038/srep40998 28102355 PMC5244412

[B5] Chang R. W. Chang C. Y. Wu M. S. Yu H. Y. Luo J. M. Chen Y. S.. (2015). Systolic aortic pressure-time area is a useful index describing arterial wave properties in rats with diabetes. Sci. Rep. 5:17293. 10.1038/srep17293 26620634 PMC4664900

[B6] Chen C. H. Nevo E. Fetics B. Pak P. H. Yin F. C. Maughan W. L.. (1997). Estimation of central aortic pressure waveform by mathematical transformation of radial tonometry pressure. Validation of generalized transfer function. Circulation 95, 1827–1836. 10.1161/01.CIR.95.7.1827 9107170

[B7] Cox R. H. (1974). Three-dimensional mechanics of arterial segments *in vitro* methods. J. Appl. Physiol. 36, 381–384. 4814310 10.1152/jappl.1974.36.3.381

[B8] Cox R. H. (1977). Effects of age on the mechanical properties of rat carotid artery. Am. J. Physiol. 233, H256–H263. 888969 10.1152/ajpheart.1977.233.2.H256

[B9] Franklin S. S. (2006). Hypertension in older people: part 1. J. Clin. Hyperten. 8, 444–449. 10.1111/j.1524-6175.2006.05113.x 16760685 PMC8109573

[B10] Gundel W. Cherry G. Rajagopalan B. Tan L. B. Lee G. Schultz D. (1981). Aortic input impedance in man: acute response to vasodilator drugs. Circulation 63, 1305–1314. 10.1161/01.CIR.63.6.1305 7226476

[B11] Kelly R. Hayward C. Avolio A. O'Rourke M. F. (1989). Noninvasive determination of age-related changes in the human arterial pulse. Circulation 80, 1652–1659. 10.1161/01.CIR.80.6.1652 2598428

[B12] Lakatta E. G. (1979). Alterations in the cardiovascular system that occur in advanced age. Fed. Proc. 38, 163–167. 153853

[B13] Lakatta E. G. Yin F. C. P. (1982). Myocardial aging: functional alterations and related cellular mechanisms. Am. J. Physiol. 242, H927–H941. 6283905 10.1152/ajpheart.1982.242.6.H927

[B14] Latson T. W. Yin F. C. P. Hunter W. C. (1987). The effects of finite wave velocity and discrete reflection on ventricular loading, in Ventricular/Vascular Coupling: Clinical, Physiological, and Engineering Aspects, ed Yin F. C. P. (New York, NY: Springer-Verlag), 354–383.

[B15] Laxminarayan S. Sipkema P. Westerhof N. S. (1978). Characterization of the arterial system in the time domain. IEEE Trans. Biomed. Eng. 25, 177–184. 10.1109/TBME.1978.326244 640704

[B16] London G. M. Pannier B. (2010). Arterial functions: how to interpret the complex physiology. Nephrol. Dial. Transplant. 25, 3815–3823. 10.1093/ndt/gfq614 20947536

[B17] Mattace-Raso F. U. S. Van Der Cammen T. J. M. Hofman A. Van Popele N. N. Bos M. L. Schalekamp M. A.. (2006). Arterial stiffness and risk of coronary heart disease and stroke: the Rotterdam Study. Circulation 113, 657–663. 10.1161/CIRCULATIONAHA.105.555235 16461838

[B18] McDonald D. A. (1974). Blood Flow in Arteries, 2nd Edn. London: Edward Arnold.

[B19] Milnor W. R. (1989). Hemodynamics, 2nd Edn. Baltimore, MD: Williams & Wilkins.

[B20] Murgo J. P. Westerhof N. Giolma J. P. Altobelli S. A. (1981). Manipulation of ascending aortic pressure and flow with the Valsalva maneuver: relationship to input impedance. Circulation 63, 122–132. 10.1161/01.CIR.63.1.122 7438386

[B21] Najjar S. S. Scuteri A. Lakatta E. G. (2005). Arterial aging: is it an immutable cardiovascular risk factor? Hypertension 46, 454–462. 10.1161/01.HYP.0000177474.06749.98 16103272

[B22] Nichols W. W. O'Rourke M. F. (2011). McDonald's Blood Flow in Arteries, 6th Edn. London: Edward Arnold.

[B23] Nichols W. W. O'Rourke M. F. Avolio A. P. Yaginuma T. Murgo J. P. Pepine C. J.. (1985). Effects of age on ventricular-vascular coupling. Am. J. Cardiol. 55, 1179–1184. 10.1016/0002-9149(85)90659-9 3984897

[B24] O'Leary D. H. Polak J. F. Kronmal R. A. Manolio T. A. Burke G. L. Wolfson S. K. Jr. (1999). Carotid-artery intima and media thickness as a risk factor for myocardial infarction and stroke in older adults. Cardiovascular Health Study Collaborative Research Group. N. Engl. J. Med. 340, 14–22. 10.1056/NEJM199901073400103 9878640

[B25] O'Rourke M. F. (1982). Vascular impedance in studies of arterial and cardiac function. Physiol. Rev. 62, 571–652. 6461866 10.1152/physrev.1982.62.2.570

[B26] O'Rourke M. F. Nichols W. W. (2005). Aortic diameter, aortic stiffness, and wave reflection increase with age and isolated systolic hypertension. Hypertension 45, 652–658. 10.1161/01.HYP.0000153793.84859.b8 15699456

[B27] O'Rourke M. F. Yaginuma T. Avolio A. P. (1984). Physiological and pathophysiological implications of ventricular/vascular coupling. Ann. Biomed. Eng. 12, 119–134. 10.1007/BF02584226 6439084

[B28] Schleicher E. D. Wagner E. Nerlich A. G. (1997). Increased accumulation of the glycoxidation product N(epsilon)-(carboxymethyl)lysine in human tissues in diabetes and aging. J. Clin. Invest. 99, 457–468. 10.1172/JCI119180 9022079 PMC507819

[B29] Sims T. J. Rasmussen L. M. Oxlund H. Bailey A. J. (1996). The role of glycation cross-links in diabetic vascular stiffening. Diabetologia 39, 946–951. 10.1007/BF00403914 8858217

[B30] Sipkema P. Westerhof N. Randall O. S. (1980). The arterial system characterized in the time domain. Cardiovasc. Res. 14, 270–279. 10.1093/cvr/14.5.270 7388858

[B31] Sutton-Tyrrell K. Najjar S. S. Boudreau R. M. Venkitachalam L. Kupelian V. Simonsick E. M.. (2005). Elevated aortic pulse wave velocity, a marker of arterial stiffness, predicts cardiovascular events in well-functioning older adults. Circulation 111, 3384–3390. 10.1161/CIRCULATIONAHA.104.483628 15967850

[B32] Van Bortel L. M. Balkestein E. J. van der Heijden-Spek J. J. Vanmolkot F. H. Staessen J. A. Kragten J. A.. (2001). Non-invasive assessment of local arterial pulse pressure: comparison of applanation tonometry and echo-tracking. J. Hypertens. 19, 1037–1044. 10.1097/00004872-200106000-00007 11403351

[B33] Wang C. H. Chang R. W. Ko Y. H. Tsai P. R. Wang S. S. Chen Y. S.. (2014a). Prevention of arterial stiffening by using low-dose atorvastatin in diabetes is associated with decreased malondialdehyde. PLoS ONE 9:e90471. 10.1371/journal.pone.0090471 24595201 PMC3942436

[B34] Wang C. H. Wu E. T. Wu M. S. Tsai M. S. Ko Y. H. Chang R. W.. (2014b). Pyridoxamine protects against mechanical defects in cardiac aging in rats: studies on load dependence of myocardial relaxation. Exp. Physiol. 99, 1488–1498. 10.1113/expphysiol.2014.082008 25239923 PMC4240468

[B35] Westerhof B. E. (2005). Blood Pressure Analysis on Time Scales From Seconds to Days (dissertation). Amsterdam: University of Amsterdam.

[B36] Westerhof B. E. Guelen I. Westerhof N. Karemaker J. M. Avolio A. (2006). Quantification of wave reflection in the human aorta from pressure alone: a proof of principle. Hypertension 48, 595–601. 10.1161/01.HYP.0000238330.08894.17 16940207

[B37] Westerhof N. Sipkema P. van den Bos G. C. Elzinga G. (1972). Forward and backward waves in the arterial system. Cardiovasc. Res. 6, 648–656. 10.1093/cvr/6.6.648 4656472

[B38] Wu E. T. Liang J. T. Wu M. S. Chang K. C. (2011). Pyridoxamine prevents age-related aortic stiffening and vascular resistance in association with reduced collagen glycation. Exp. Gerontol. 46, 482–488. 10.1016/j.exger.2011.02.001 21316441

[B39] Wu M. S. Chang C. Y. Chang R. W. Chang K. C. (2012). Early return of augmented wave reflection impairs left ventricular relaxation in aged Fisher 344 rats. Exp. Gerontol. 47, 680–686. 10.1016/j.exger.2012.06.006 22750485

[B40] Yin F. C. P. (1980). The aging vasculature and its effects on the heart, in The Heart in Old Age: Its Function and Response to Stress, ed Weisfeldt M. L. (New York, NY: Raven), 137-217.

